# New insights into Human Hematopoietic Stem and Progenitor Cells via Single-Cell Omics

**DOI:** 10.1007/s12015-022-10330-2

**Published:** 2022-03-22

**Authors:** Yawen Zhang, Yaojin Huang, Linping Hu, Tao Cheng

**Affiliations:** 1 State Key Laboratory of Experimental Hematology, National Clinical Research Center for Blood Diseases, Haihe Laboratory of Cell Ecosystem, Institute of Hematology & Blood Diseases Hospital, Chinese Academy of Medical Sciences & Peking Union Medical College, 288 Nanjing Road, Tianjin, 300020 China; 2 Department of Hematology, Jiangsu Province Hospital, The First Affiliated Hospital of Nanjing Medical University, Nanjing, 210000 China

**Keywords:** Hematopoietic stem and progenitor cells, Single-cell omics, Hematopoiesis, Hematopoietic malignancies

## Abstract

Residing at the apex of the hematopoietic hierarchy, hematopoietic stem and progenitor cells (HSPCs) give rise to all mature blood cells. In the last decade, significant progress has been made in single-cell RNA sequencing as well as multi-omics technologies that have facilitated elucidation of the heterogeneity of previously defined human HSPCs. From the embryonic stage through the adult stage to aging, single-cell studies have enabled us to trace the origins of hematopoietic stem cells (HSCs), demonstrating different hematopoietic differentiation during development, as well as identifying novel cell populations. In both hematological benign diseases and malignancies, single-cell omics technologies have begun to reveal tissue heterogeneity and have permitted mapping of microenvironmental ecosystems and tracking of cell subclones, thereby greatly broadening our understanding of disease development. Furthermore, advances have also been made in elucidating the molecular mechanisms for relapse and identifying therapeutic targets of hematological disorders and other non-hematological diseases. Extensive exploration of hematopoiesis at the single-cell level may thus have great potential for broad clinical applications of HSPCs, as well as disease prognosis.

**Graphical abstract:**

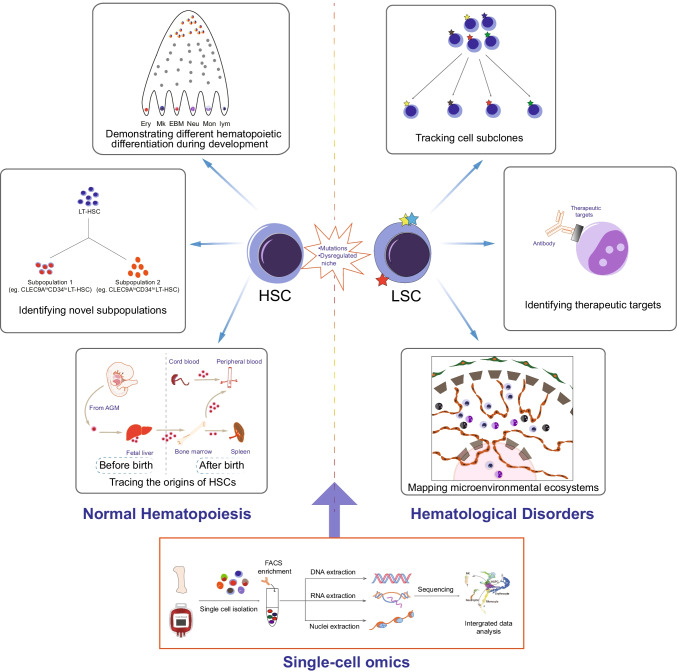

## Introduction of human hematopoietic stem and progenitor cells

The hematopoietic system is one of the most highly regenerative tissues, which is composed of billions of erythrocytes, platelets, myeloid cells, innate and adaptive immune cells. It is involved in blood cell formation, coagulation function, immune response, and other physiological processes. Residing at the hierarchical top of hematopoiesis, hematopoietic stem and progenitor cells (HSPCs) give rise to all mature blood cells [1], and even a single hematopoietic stem cell (HSC) can produce long-term and multipotent reconstitution of the entire blood system [2, 3].

In 1961, Till and McCulloch first demonstrated the existence of multipotent HSCs through observing colony-forming units in the spleen following in vivo lethally irradiated transplantation [4]. The subsequent discovery of surface markers to purify HSCs, coupled with fluorescence-activated cell sorting (FACS), made it possible to isolate HSC and progenitor populations. In human hematopoiesis, CD34, which is expressed on a minority of blood cells, was the first human HSPC surface marker discovered in 1984 [5] and was widely used in clinical HSC transplantation [6, 7]. However, some studies showed that CD34^−^ HSCs in human cord blood (CB) may exist [8–10]. To date, surface markers including CD38 [11, 12], CD45RA [13], CD90 [14], CD49f, rhodamine-123 [3], GPI80 [15], etc. have been applied to purify human HSCs in different laboratories. Similarly, multi-, oligo- and unipotent progenitor cells also have been separated by different surface markers [16]. These immunophenotype-defined assays have been commonly used and have contributed greatly to the research of hematopoiesis over the past decades. However, it is worth noting that due to alterations of surface marker expression after culture or transplantation [17], immunophenotype may not be an accurate representation of the true HSPCs in all scenarios.

Functional assays such as in vivo xenograft and in vitro colony formation are the “gold standard” to confirm the existence of human HSCs and progenitor cells [16]. HSCs are characterized by self-renewal and multipotency, while progenitor cells are oligo- or unipotent due to their insufficiency to differentiate into all blood lineages and lack of self-renewal ability [18, 19]. The classical human hematopoietic hierarchy is usually described as a tree-like model, which starts with HSCs, followed by multipotent progenitors (MPPs), downstream progenitors and mature cells [16]. The first lineage decision followed by MPPs to segregate myeloid/erythroid from myeloid/lymphoid fates coincides with the population of common myeloid progenitors (CMPs) and multi-lymphoid progenitors (MLPs) [20–22]. A second lineage decision occurs allowing CMPs to form megakaryocyte-erythroid progenitor progenitors (MEPs) and granulocyte-monocyte progenitors (GMPs). These progenitors differentiate into erythrocytes, megakaryocytes, granulocytes, monocytes, T, B, NK, and dendritic cells. Although the classical human hierarchical model has been a great tool to understand hematopoiesis, it should be noted that this model was established by immunophenotyped populations and functional assays of bulk cells.

The heterogeneity of immunophenotype-defined HSPC populations has restricted further understanding of human hematopoiesis in normal and disease states, thus, to overcome these limitations, researchers have turned to study HSPCs at more precise levels, even at the single-cell level. From a single-cell perspective, in vitro single-cell culture cannot fully display all blood lineages due to technical limitations and differentiation bias caused by different culture systems. Moreover, the insufficient cell numbers (or just one cell) make the in vivo xenograft experiments extremely difficult. Therefore, neither immunophenotype nor functional experiments can entirely define true HSPCs. With recent advances in single-cell technologies, single-cell transcriptomics became another powerful means to recognize hematopoiesis as well as HSPCs within the past few years. Through this new technology, new subpopulations of HSPCs [23], a revised roadmap of the hematopoietic system [24] and malignancy hierarchies relevant to disease progression [25] have been proposed by different groups. Our group has also detailed a single-cell landscape of the human blood cells for providing a comprehensive reference of hematopoiesis [26]. In conclusion, functional, immunophenotypic and transcriptomic (FIT)-defined HSPCs represent future directions for advancing the understanding of hematopoiesis [27].

## Advances in single-cell technologies

### Single-cell transcriptomics

**Fig. 1:**
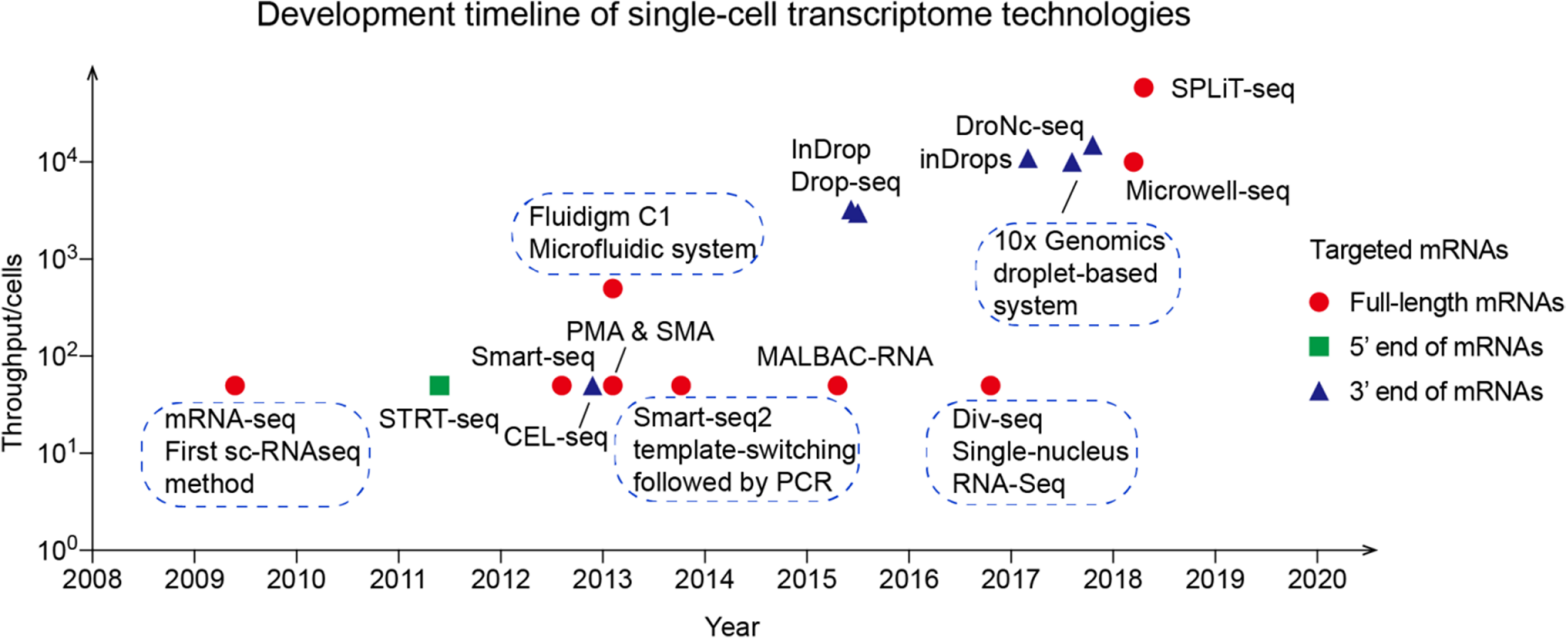
Development timeline of single-cell transcriptome technologies

Since Tang et al*.* [28] first established the single-cell mRNA-Seq method in 2009, single-cell transcriptomic technologies have enabled great progress in the last ten years, owing to the capability of sequencing at increasing throughput and decreasing cost. Single-cell RNA sequencing (scRNA-seq) is composed of several steps: single-cell capture, RNA obtainment, cDNA amplification, and library construction. Wu et al*.* [29] used a microfluidic-based system for cell capture, lysis, and pre-amplification in one chip. In 2015, scientists developed two methods, separately termed Drop-seq [30] and InDrop [31], which combined barcoded primer beads and droplets to achieve throughput reaching thousands of individual cells. With the development of droplet-based assays such as 10 × Genomics [32] and inDrops [33], tens of thousands of single cells could be captured per sample. Subsequently, Guo’s laboratory developed Microwell-Seq [34] using microwell arrays constructed from agarose and barcoded beads, which drastically reduced the cost. SPLiT-seq [35] utilized four rounds of barcoding strategies to label the cellular origins of RNA. Each round of barcoding could append different labels to cDNA, ultimately labeling over 1 million cells. Figure 1 summarizes the recent advances in single-cell technologies.

To achieve high efficiency and low bias of single-cell sequencing, researchers developed various assays: STRT-seq [36] and Smart-seq/Smart-seq2 [37–39] used template-switching technology followed by PCR for full-length cDNA; others like CEL-Seq [40], Phi29-mRNA amplification (PMA), SRP-mRNA amplification (SMA) [41] and Multiple Annealing and Looping-Based Amplification Cycles (MALBAC) [42] adopted linear amplification or other methods for cDNA. However, most of the aforementioned assays need freshly prepared and live single-cell suspensions to extract cytoplasmic RNA, hence Div-Seq [43] and DroNc-Seq [44] were developed to profile RNA from preserved or undissociated samples. Although there is still some room for improvement in single-cell transcriptomic technologies, it will have broad potential applications in biological and clinical research.

### Single-cell multi-omics

Although single-cell transcriptomics is a great tool to quantify expression variability between individual cells, it is not possible to use it to explore the correlation of genotype, phenotype, gene expression, or even chromatin structure in single cells. It was a great challenge for scientists to integrate transcriptomics, genomics, proteomics and epigenomics in a single cell. However, in 2015, DR-Seq [45] and G&T-seq [46] made it possible to separate and sequence genomic DNA and full-length mRNA from single cells. Moreover, DNTR-seq [47] can combine whole genome DNA and mRNA sequencing at the single-cell level simultaneously. Tang’s laboratory described scTrio-seq [48], which can simultaneously obtain the single-cell transcriptome, DNA methylome, and genomic copy-number variations. In CITE-seq [49] and REAP-seq [50], cells labeled with antibodies conjugated to DNA barcodes and cellular protein and transcriptome can be measured simultaneously. In addition to revealing phenotypic differences and heterogeneity of cell populations, these methods enable large-scale immunophenotyping of dozens to hundreds of antibodies. Greenleaf's laboratory combined chromatin accessibility assays with gene expression data at the single-cell level to portray regulatory features in the human hematopoietic system [51]. Recently, his laboratory integrated protein quantification, gene expression, and chromatin accessibility to resolve molecular features of patients with mixed-phenotype acute leukemia (MPAL) [52]. Recently, a single-cell multi-omics assay has been developed by 10 × genomics in individual nucleus. Instead of using algorithmic integration method, this technology enables to perform RNA and ATAC sequencing simultaneously in single cells. This would be able to simultaneously investigate how chromatin accessibility and RNA expression determine cell fate in individual cells [53–55]. Single-cell multi-omics can provide sufficient information of cell heterogeneity, cellular subpopulations, epigenetic transitions, and the cell regulation state in the past and future. Thus, the correlation between genotype and phenotype could be captured by single-cell multi-omics. Methods for processing the single-cell multi-omics sequencing data usually include graph-based learning models and unsupervised learning methods [56]. Deep learning methods (e.g. Convolutional Neural Network (CNN) [57], Recurrent Neural Network (RNN) [58] and transformer model) have great potential in processing multi-omics sequencing which increased the data-processing performance. Recently, Simon Haas [59] et al*.* generated a single-cell proteo-genomic reference map, which linked the expression of 197 surface markers to cellular identities and biological processes across main hematopoietic cell types of BM and peripheral blood from human adults, aged and AML patients. Moreover, they developed computational tools that enable the automatic design of high-throughput cytometry schemes to isolate the molecularly defined cell state from blood and BM. However, most integrating data from DNA, RNA, or ATAC are not from the same cell. We suggest that the integration of these single-cell sequencing datasets should be used cautiously in hematological studies. Moreover, single-cell multi-omics sequencing usually generated a large number of heterogeneous data including different numbers of distributions, variables and diverse data modalities. Thus, developing a method to excavate data-specific information and to use different molecular layers information at the same time is crucial for data processing [56]. In conclusion, single-cell multi-omics could provide more insight into regulatory dynamics both in normal and disease conditions, although new technologies with low cost and high throughput are still needed.

## Applications of single-cell omics in normal hematopoiesis and diseases

### Embryonic hematopoiesis

The development of hematopoiesis is a complex process that switches from different organs in the embryonic period [60, 61]. During the prenatal stage, human HSCs first occur in the aorta-gonad-mesonephros (AGM), later in the yolk sac, fetal liver, and then finally migrate to bone marrow (BM). Additionally, some previously unappreciated sites like placenta [62, 63], umbilical arteries [64], and embryonic head [65] have been demonstrated to harbor HSCs in mice. Due to technical limitations and human sample rarity, as well as a rare number of HSC at the embryonic stage, it has been difficult to explore the precise origin of HSCs and the immune system. However, through scRNA-seq, Liu’s laboratory first established a gene expression atlas of HSC generation in the AGM region. HSC-primed hemogenic endothelial cells (HECs), the origin of HSPCs, were defined transcriptomically and could be enriched tenfold via the surface marker CD44. EMCN, PROCR, and RUNX1T1 were overexpressed at hemogenic fate choice from arterial endothelial cells via HSC-primed HECs to HSPCs [66]. To further identify the molecular mechanisms for promoting HSC emergence, Crosse et al*.* [67] applied spatial transcriptomics to compare the gene expression differences in dorsoventral polarized signaling in the aorta. A subpopulation of aortic endothelial cells, which had downregulated aortic signatures and may associate with HSPC emergence, was predicted. Also, endothelin 1 was found to be an important regulating factor of HSC development. Another study mainly focused on definitive hematopoiesis in fetal liver, in which over 200,000 single cells between 7 and 17 post-conception weeks (PCW) from liver, skin, kidney and yolk sac were sequenced to explore the human blood and immune cell developmental trajectory. Twenty-seven major cell clusters were transcriptomically defined in fetal liver and validated by morphology and imaging. Also, fetal skin was demonstrated as a physiological erythropoietic tissue and HSPCs in fetal liver showed decreased erythroid differentiation potential during gestation with functional validation [68]. Recently, hematopoietic development in human fetal BM was detailed from 12 to 19 PCW from nine normal fetuses. The whole blood and immune system were established during the early second trimester. Also, alterations of gene expression and cellular composition between fetal liver and fetal BM were revealed [69]. Integrative analysis of both scRNA-seq and scATAC-seq highlighted epigenetic priming of HSC/MPPs before lineage commitment [70], which was divergent from the conventional opinion that transcriptional priming emerged first. Although there have been many studies on the development of HSPCs during the fetal period, a more detailed molecular analysis of the dynamics of HSPCs as well as determining when transplantable HSCs emerge in fetal BM remains to be precisely investigated.

The development and origin of the immune system have also been revealed at the single-cell level in the human embryo. Macrophage is a regulator which is essential to tissue development and homeostasis. Bian et al*.* [71] comprehensively characterized macrophage development during embryogenesis and found a new cell population of yolk sac-derived myeloid-biased progenitors via functional validation. This could be crucial for the diagnosis and treatment of the disease associated with the new population. T and B cells are major components of human adaptive immunity. Thus, decoding their lymphopoiesis at the fetal stage not only provides insights into the physiological development of lymphopoiesis, but also helps to understand the etiology of related blood diseases. Zeng et al*.* [72] portrayed early T lymphopoiesis and thymus organogenesis at the human embryonic stage at single-cell resolution. A new subset of early thymic progenitors was defined, which shared transcriptional similarity with thymus-seeding progenitors in the fetal liver. Also, pre-thymic lymphoid progenitors were demonstrated in the AGM region. O’Byrne et al*.* demonstrated several B-cell progenitor populations by constructing the human fetal B-lymphocyte development hierarchy in fetal BM and liver. The PreProB-progenitor was confirmed as the first B-lymphoid–restricted progenitor and upstream of ProB-progenitors at the fetal stage [73]. Meanwhile, a series of studies focused on other cell population like megakaryocyte [74] has also been published.

In summary, single-cell technologies have greatly assisted the investigation of the detailed developmental processes underlying embryonic hematopoiesis and prenatal disease initiation. It may give us more information about hematopoietic development. In the future, we might be able to improve HSC reconstitution in the patient with delayed engraftment, and manipulate the lineage bias of progenitor cells for cell therapy.

### Adult hematopoiesis

After the embryonic stage, BM is the most important microenvironment for maintaining hematopoiesis. As estimated by somatic clonal dynamics, there are approximately 50,000–200,000 HSPCs that contribute to white blood cells in adult BM [75]. Using scRNA-seq, several groups constructed the transcriptional landscape of human HSPCs in BM or CB [24, 76, 77]. A comprehensive human hematopoietic landscape constructed by single-cell transcriptome and chromatin accessibility revealed the differentiation trajectories of human hematopoiesis [51]. The lncRNAs expression profile of human HSPCs has also been generated [78]. Additionally, immunophenotype-defined stem and progenitor populations have been proven to be heterogeneous according to single-cell omics. Combining CD34 and CLEC9A expression, HSCs were divided into two groups: CLEC9A^high^CD34^low^ HSCs with multipotency and more quiescence and CLEC9A^low^CD34^high^ HSCs with myeloid and lymphoid potential [23]. Another study pointed out that compared to CD33^−^ HSCs, CD33^+^ HSCs have more durable regenerative potential [79]. For the progenitors, the immunophenotypic CMPs and GMPs were reported to be heterogenous by an integrated analysis of scRNA-seq and scATAC-seq. GMPs were further divided into three sequential populations with distinct myeloid developmental levels [51]. Karamitros et al. showed via transcriptional and functional validation that lymphoid primed multi-potential progenitors (LMPPs), GMPs and MLPs were heterogeneous populations. Although most of them were unipotent, there were still a few of these progenitors that showed bi- and multipotency [21].

The molecular mechanisms of fate decision that play an important role in hematopoiesis have also been investigated. Recently, Lu et al*.* [80] demonstrated that the fate decision of MEPs was affected by cell cycle speed. When the cell cycle speed was increased, MEPs were biased towards erythrocyte specification, and when the cell cycle speed was decreased, MEPs were biased towards megakaryocyte differentiation. According to the upregulation of transcription factor GATA2, it is plausible that eosinophils/basophils/mast cells may have common ancestors with erythrocytes in human hematopoiesis, and this has been proved in the mouse model [81]. Taken together, more precise definition of subsets from HSPCs could be acquired through single-cell omics, thus leading to a better understanding of hematopoiesis.

**Fig. 2:**
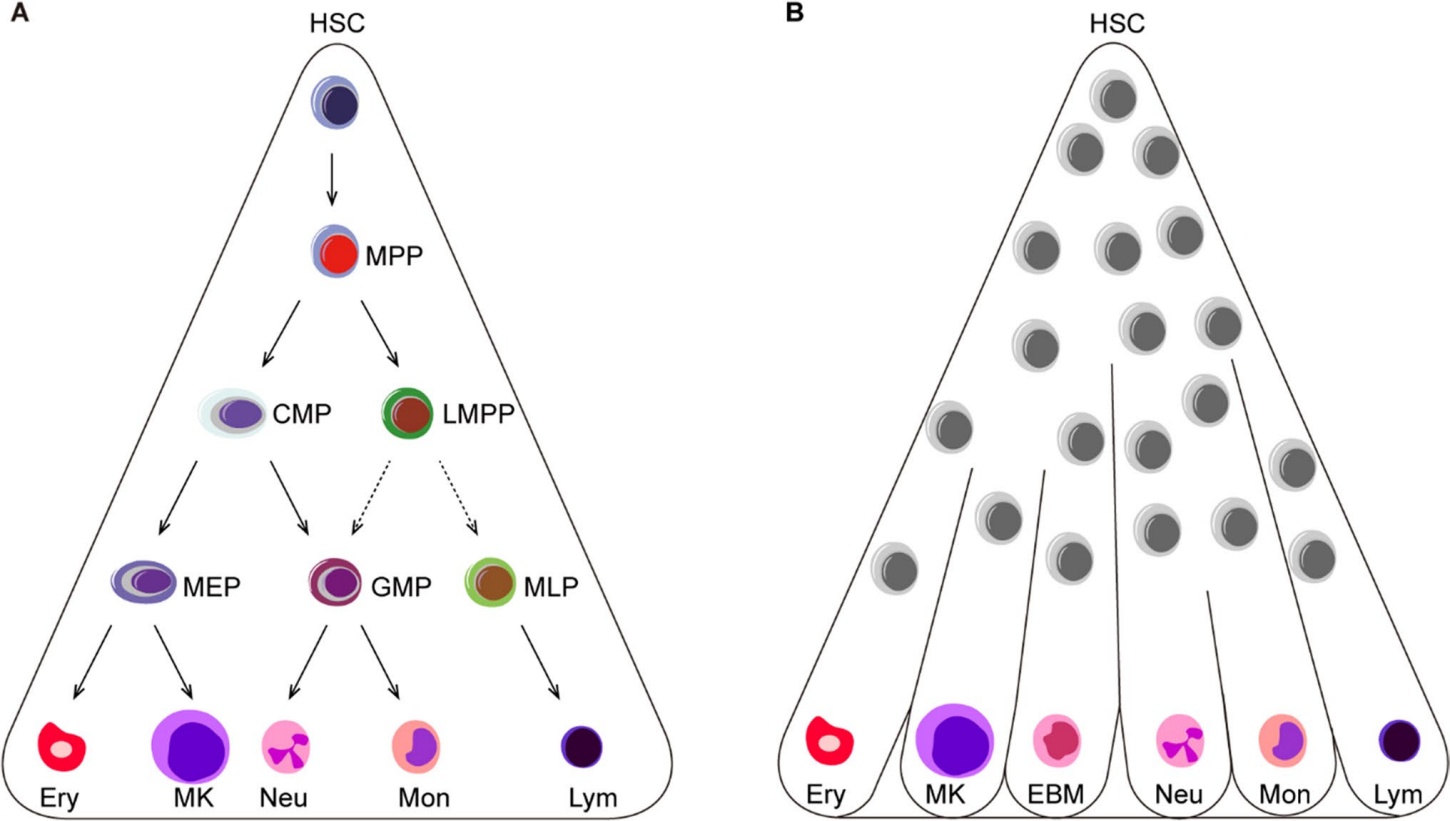
Human hematopoietic hierarchy models. **A** The classical tree-like human hematopoietic hierarchy. HSCs differentiate into multi-, bi- and unipotent progenitor cells, and progenitors give rise to all mature blood cells. **B** The continuum human hematopoietic differentiation model. Continuum of low-primed undifferentiated HSPCs sit at the hierarchical top of hematopoiesis and differentiate into unilineage-restricted cells. Ery, erythrocyte; MK, megakaryocyte; EBM, eosinophil, basophil and mast cells; Neu, neutrophils; Mon, monocyte; Lym, lymphocyte

To date, several studies have challenged the classical tree-like hierarchy (Fig. 2) [21, 24, 76, 82]. Recent research provided a continuum differentiation model through a comprehensive overview of human BM HSPCs by single-cell technologies [24]. A continuum of low-primed undifferentiated HSPCs was at the hierarchical top of hematopoiesis, and there were no specific progenitor populations like CMPs during the period from HSCs to unilineage-restricted cells. This view was confirmed by another single-cell study performed in human CB HSPCs with unbiased computational analysis, in which there were intermediate stages closely related to stem cell populations without distinct fate choices [76]. Distinct differentiation potentials of LMPPs, MLPs, and GMPs also confirmed that a continuum of progenitors differentiate downstream of stem cells [21]. Additionally, the differentiation model of adult hematopoiesis is different from that at the fetal stage. Dick’s group demonstrated previously through in vivo and in vitro functional assays that immunophenotypic MPPs, CMPs and MEPs are heterogeneous populations, and that there is a differentiation shift from fetal to adult: many HSPCs were multipotent at the fetal stage, while stem cells show multipotency and progenitors were unipotent at the adult stage [83]. However, although this study focused on adult and fetal stage, alterations of human HSPCs in the period after birth to adult still requires further research.

In summary, single-cell omics plays an important role in understanding human hematopoiesis. It is of critical importance to integrate the tools of immunophenotyping, functional validation, and transcriptomics to properly define HSPCs and gain a better understanding of the blood system.

### HSPC niche

Single-cell omics are great tools to explore HSPC niche, which represents a three-dimensional space comprising several types of components interact with and regulate HSPCs [84, 85]. Recently, spatial transcriptomic technologies provided new perspectives in exploring HSPC niche both in human and mouse models [67, 86–88]. In short, there are four types of spatial transcriptomics, including computational methods for spatial reconstruction, laser capture microdissection (LCM)-based methods, in situ RNA imaging methods and in situ sequencing methods. Crosse et al*.* combined spatial, population, and single-cell transcriptomics to interrogate signaling in the human HSC embryonic niche. By using LCM coupled with RNA sequencing, molecular differences between orsal-ventral and the dorsal aorta were carefully explored. And it focused on cell layers close to intra-aortic hematopoietic cell clusters (IAHCs) formation. By analysis of the ventrally polarized molecular landscape, endothelin 1 was identified as an important secreted regulator for human HSC development. Interestingly, cardiac EGF pathway was enriched next to developing IAHCs/HSCs, and endothelin 1, secreted by ventral portions, was found to promote the development of HSCs. However, studies related to the niche of human adult hematopoiesis were still lacking [67]. The immune microenvironment in hematopoietic malignance and diseases could be the future directions of the spatial transcriptomics.

### Aging and benign diseases

Hematopoietic aging in humans always manifests a high risk of myeloid malignancies and functional decline of HSCs. With an integrative characterization of epigenomic and transcriptomic changes, Adelman et al*.* [89] portrayed the map of regulatory elements in normal human HSC during aging. The epigenetic reprogramming of human HSCs occurred with age, which was particularly evident at active enhancers. For example, KLF6 was identified as not only the most downregulated transcription factor with aging, but also as aberrantly methylated in acute myeloid leukemia (AML). This indicated that age-related dysfunction of the human HSC may also be mediated by dysfunction of these regulatory elements, which may increase the risk of malignant transformation.

In benign diseases like BM failure (BMF) syndromes and aplastic anemia (AA), chromosomal abnormalities were frequently observed and monosomy 7 in BMF was associated with poor clinical outcomes. Young’s group [90] used scRNA-seq to distinguish aneuploid cells from diploid cells within the HSPCs of BMF patients. The aneuploid cells in this study were demonstrated to exhibit downregulation of genes involved in immune response and DNA stability. Recently, our group analyzed the relationship between HSPCs and T cells in AA patients through single-cell transcriptome. Cell-type-specific ligand-receptor interactions were revealed as potential factors for the continuous destruction of HSPCs by T cells [91].

Though single-cell omics have been applied to explore numerous hematopoietic disorders, a few studies related to rare disorders of the hematopoietic system have not been reported. Diamond-Blackfan anemia (DBA) is a rare ribosomopathy. Patients who develop DBA usually receive limited therapeutic options. Using single-cell transcriptomes, Deena et al*.* [92] presented an unbiased charting of erythropoiesis in RPS-DBA and RPL-DBA and defined genotype–phenotype correlations in DBA. Moreover, they found compensatory stress erythropoiesis in RPL-DBA exhibited altered glucocorticoid molecular signature, including reduced ZFP36L2 expression, leading to milder anemia and improved corticosteroid response. Therefore, ZFP36L2 may become candidate therapeutic targets for failing erythropoiesis.

Paroxysmal nocturnal hemoglobinuria (PNH), which is a rare clonal hematopoietic stem cell disorder that manifests with hemolytic anemia, thrombosis and peripheral blood cytopenia. PNH begins with the expansion of a HSC that has a severe deficiency or absence for GPI, a glycolipid moiety that anchors > 150 different proteins to the cell surface which deficiency in virtually all PNH cases is the result of a somatic mutation in PIGA [93]. The mechanisms leading to PNH stem cell clonal expansion and dominance remain unclear. Therefore, research into this disease at single cell level is needed.

On the other hand, although the increased throughput and decreased cost of single-cell omics sequencing emerged recently, it is still a problem how to change it from descriptive research towards mechanistic insights and/or precision medicine. Further research is needed to explore physiological regulation of human hematopoietic development and discover potential gene targets for directed therapy.

### Malignancies

A series of mutations and/or epigenetic events in HSCs can lead to the occurrence of malignant hematopoiesis. In this section, we will discuss the application of single-cell omics to malignant hematopoiesis (Table 1), which has provided new insights into mechanisms of clonal evolution, drug resistance, and disease relapse.

**Table 1:** Single-cell omics on malignant hematopoiesis

Disease	Methodology	Sample input	Ref
AML	ScRNA-seq (Seq-Well) and single-cell genotyping	30,712 cells from 16 AML patients and 7,698 cells from 5 healthy donors	[25]
	ScATAC-seq	71 LSCs and 42 blast cells from two AML patients 88 normal monocytes and 94 LMPPs isolated from healthy donors	[99]
	ScRNA-seq (10 × Genomics)	Peripheral blood mononuclear cells from one older AML patient at baseline and after two and four days of therapy	[101]
	ScRNA-seq (MutaSeq)	618 to 1,430 cells per patient from 4 AML patients	[96]
	ScDNA-seq	735,483 cells from 154 AML samples (140 from BM and 14 from peripheral blood) of 123 patients	[102]
	ScDNA-seq	740,529 cells from 146 samples of 123 patients with myeloid malignancies (clonal hematopoiesis, MPN or AML)	[103]
ALL	ScDNA-seq and scRNA-seq (10 × Genomics)	1,332 leukemic cells from 4 childhood T-ALL patients 8,296 cells from 4 childhood T-ALL	[105]
	Single-cell targeted DNA sequencing	108,188 cells from 25 samples (12 from BM and 13 from peripheral blood) of 8 T-ALL patients	[106]
	Mass cytometry analysis	BM aspirates from 60 patients with BCP-ALL and five healthy donors	[104]
	ScRNA-seq (5′ 10 × Genomics and TCR V(D)J)	25,386 CD19^+^ cells and 24,157 CD19^−^CD3^+^ cells from 4 samples from B-ALL patients before blinatumomab treatment (two responders and two non-responders)	[107]
	ScRNA-seq (10 × Genomics)	53,447 cells from BM samples of 7 B-ALL patients (CD19^+^ cells: CD19^−^CD45^+^ cells, radio = 1:5) and 4 healthy controls (CD45^+^ cells)	[109]
MPAL	ScRNA-seq (CITE-seq) and scATAC-seq	CITE-seq: 35,882 cells from 6 healthy donors, 18,056 cells from 6 MPAL patients ScATAC-seq: 35,038 cells from 10 healthy donors, 35,423 cells from 6 MPAL patients	[52]
CML	Sc-qPCR	2,151 single LSCs from 22 CP-CML patients 5 age-matched healthy controls	[111]
	ScRNA-seq (Smart-Seq2)	Over 2,000 stem cells from CML patients	[112]
	ScRNA-seq (Fluidigm C1)	150 LSC-CD93^+^cells, 150 LSC-CD93^−^ cells from 2 CP-CML patients	[113]
	ScRNA-seq (Smart-Seq2)	144 CML-stem cells and 144 HSCs from 3 CML patients	[114]
	ScRNA-seq (Smart-Seq2)	245 cells from 16 CML patients	[115]
CLL	MscRRBS ScRNA-seq (Smart-Seq2)	831 normal B cells from six healthy donors, and 1,821 cells from 12 primary IGHV mutated and unmutated CLLs	[118]
	Single-cell targeted DNA sequencing ScRNA-seq (Smart-Seq)	1,152 cells from the 5 CLL patients 96 cells from 4 CLL patients 384 cells from each of 7 CLL patients and from normal CD19^+^ B cells	[119]
	ScRNA-seq (inDrops)	1,035–3,751 cells per sample from 4 patients	[120]
MDS	Single cell targeted sequencing	Sorted stem and blast populations with selected mutations in 7 MDS patients who had later progressed to AML	[122]
	ScDNA-seq	Mononuclear cells in 21 BM samples from 8 patients with MDS and progression to AML	[123]
MPN	scRNA-seq (3’-TARGET-seq)	752 HSCs from 7 JAK2-V617F^+^ essential thrombocythemia patients, 359 HSCs from 6 JAK2-V617F^+^ patients posttreatment and 485 from 7 healthy controls	[124]
	ScRNA-seq (10 × Genomics)	93,157 lin^−^CD34^+^ HSPCs from 15 patients with myelofibrosis and 42,772 lin^−^CD34^+^ HSPCs from 6 healthy donors	[125]
	ScRNA-seq (10 × Genomics)	52,127 HSPCs from 7 newly diagnosed patients with polycythemia vera (n = 3), essential thrombocythemia (n = 4) and healthy controls (n = 2)	[126]
MM	ScRNA-seq (Mars-seq)	20,586 single plasma cells from the BM and 3,540 single plasma cells from 11 control individuals and 29 newly diagnosed MM patients	[128]
	Single-cell targeted qRT-PCR	528 pre-treatment single cells from 11 myeloma cell lines and 418 single cells from 8 drug-naive MM patients	[129]
	ScRNA-seq (10 × Genomics)	17,267 plasma cells and 57,719 immune cells from 29 samples with 14 MM patients at different disease stages	[130]

### Acute leukemia

AML is an aggressive hematological malignancy which leads to a poor clinical outcome. Most patients die from the disease relapse that is related to clonal evolution at the level of cytogenetics [94, 95]. ScRNA-seq is well-suited to characterize AML heterogeneity, illustrate AML tumor ecosystems, and validate subclones. Hence, many studies have focused on distinguishing AML blasts from normal cells [25, 96, 97]. Galen et al*.* [25] integrated single-cell transcriptomics and genomic data of AML samples and healthy donors to distinguish malignant from normal cells in AML samples. They identified six malignant cell types along the axis of HSC to myeloid differentiation and revealed a striking consistency between developmental hierarchy and tumor genetics. Gene expression analysis revealed that patients with higher HSC/progenitor-like signals exhibited significantly worse clinical outcomes than patients with higher expression of GMP-like genes. These results are in accordance with the existence of leukemia stem cells (LSCs), which have been proved to be capable of initiating and maintaining leukemia and linked to poor prognosis, therapy resistance and high rate of relapse in AML [98]. Velten et al*.* [96] revealed that LSCs, pre-LSCs and normal HSCs could be distinguished through single-cell transcriptomics due to genomic and mitochondrial mutations. Additionally, Corces et al*.* [99] demonstrated that Hox-mediated chromatin accessibility loss was the most common defect in pre-leukemic HSCs (pHSCs). Losing HOX factors may lead to differentiation defects like those observed in pHSCs and contribute to an evolutionary advantage. Xu et al*.* [100] also added the evidence of heterogeneity of the pHSCs population and revealed that the pHSCs burden may reflect the diversity of pHSCs and predict poor prognosis. Moreover, scRNA-seq studies revealed the disease dynamics on older AML patients before and after exposure to the B-cell lymphoma 2 inhibitor venetoclax and azacytidine, in which there were no changes in normal hematopoietic cells, whereas cell blasts were rapidly depleted [101]. These studies also showed that therapeutic interventions eradicate LSCs in AML patients by disrupting metabolic mechanisms that drive energy metabolism, providing insight into clinical use in patients with historically poor outcomes. Recently, clonal evolution in AML was revealed by different groups [102, 103]. Through single-cell DNA sequencing of 146 samples from 123 patients with myeloid malignancies, Miles et al*.* [103] found that AML was dominated by a few clones which mostly cover co-occurring epigenetic mutations. In contrast, signaling mutations often occurred in subclones more than one time. Such studies at the single-cell level could thus provide further crucial information on initiation and progression for AML.

To date, there have been limited such studies regarding acute lymphoblastic leukemia (ALL) initiation and progression in comparison with AML [104, 105]. Cools’ laboratory used targeted single-cell sequencing of total BM cells and CD34^+^CD38^−^ multipotent progenitor cells to reveal the genetic basis of disease initiation in T-cell ALL (T-ALL). In half of the cases, mutations could be detected in CD34^+^CD38^−^ cells, which proved that the order of mutation acquisition in T-ALL may initiate from the multipotent progenitor cells [105]. Another study described clonal evolution at diagnosis and during treatment in T-ALL patients in which a minor clone evolved to the major clone at the advanced stage of disease[106]. Additionally, through single-cell mass cytometry and machine learning, individual B cell precursor ALL (BCP-ALL) cells were mapped to normal B-cell trajectories and pre-pro-B cell to pre-BI cell transition was expanded. A new model, termed ‘developmentally dependent predictor of relapse’ was developed to predict the risk of relapse at diagnosis in BCP-ALL patients [104]. In addition, some studies have applied single-cell technologies to focus on effects of therapies for B-ALL, including blinatumomab [107] and chimeric antigen receptor T cell therapy [108]. Immune microenvironment re-modeling has also been characterized via single-cell technologies during B-ALL progression, in which low non-classical monocytes frequency implied a high survival rate in B-ALL [109]. Furthermore, single-cell omics has been used to reveal epigenetic alternations in mixed phenotype acute leukemia (MPAL), in which RUNX1 may act as a potential oncogene, resulting in poor survival of MPAL patients [52].

Taken together, such studies have offered new insights into heterogeneity, clonal evolution and cellular hierarchies during disease initiation and progression of acute leukemia at the single-cell level that could not be unraveled by bulk analysis. In future studies, determining the precise kinetics of clonal evolution from diagnosis and remission to relapse and response to new therapies would be promising avenues for the further application of single-cell methodologies.

### Chronic leukemia

Chronic myeloid leukemia (CML) is primarily caused by the oncogenic fusion protein BCR-ABL, and tyrosine kinase inhibitors (TKIs) have shown potent efficacy in the treatment of CML. Nonetheless, many patients relapse after treatment, mainly due to selective resistance of CML stem cells (CML-SCs) to TKIs [110, 111]. Recently, a variety of studies focused on this issue have sought to identify the molecular mechanisms of relapse as well as therapeutic targets at the single-cell level [112–115]. Giustacchini et al*.* developed a new method, which combined high-sensitivity mutation detection with whole transcriptome analysis in the same cell, to analyze more than 2,000 CML-SCs from patients. Through this method, BCR-ABL^+^ SCs can be separated from BCR-ABL^−^ SCs, and a subpopulation of BCR-ABL^+^ SCs resistant to TKIs was identified, which may become a putative therapeutic target. [112]. In addition, several surface markers were identified to enrich the TKIs-resistant CML-SCs, such as CD26 and CD93 [111, 113]. Another study confirmed that PIM2, a serine/threonine kinase, was required for imatinib mesylate (one of the TKIs) resistance in CML-SCs. A combination of imatinib mesylate with a PIM inhibitor can increase CML-SCs apoptosis, decrease colony formation, and prolong survival of the CML mouse model, without obvious side effects on HSCs [114]. Additionally, the bone morphogenetic protein receptor type-1B and Jak2/Stat3 signaling were activated in persisting and dormant SCs, and targeting these signals could affect CML-SCs in the BM niche [115].

Chronic lymphocytic leukemia (CLL) is a complex heterogeneous cancer with substantial genetic diversity and evolution during disease progression and treatment [116–118]. Combining whole transcriptome analysis with genomic information at the single-cell level could help to elucidate the underpinnings of CLL disease initiation and development. Wang et al*.* [119] utilized single-cell genomic and transcriptome analysis to reveal that mutations in LCP1 and WNK1 may be novel drivers of CLL, and that there was a high degree of genetic complexity in each CLL. This phenomenon was also observed epigenetically by another study from Landau’s laboratory, in which multiplexed single-cell reduced representation bisulfite sequencing was employed to identify the lineage history and evolution accompanying the therapy of CLL, demonstrating disease heterogeneity at the epigenetic level [118]. In addition, a study of the dynamics of relapse in CLL patients after allogeneic HSC transplantation revealed that later relapses showed accelerated epigenetic alterations in comparison to early relapses. These results provided new evidence of the molecular kinetics of relapse in CLL patients [120].

### Other malignances

Myelodysplastic syndrome (MDS) progress to AML in approximately one-third of patients. A series of studies showed that MDS originated from a small group of disease-induced HSCs, which was sustained and expanded by conventional therapy and became a major factor in disease progression and relapse [121]. However, the cellular origins and mechanisms of malignant transformation from MDS to AML have not been clearly defined. Chen et al*.* [122] performed single-cell sequencing to identify stem cell and blast populations of MDS and matched AML, and found that the MDS stem cells had a higher subclonal mutation complexity than the blast cells. Also, a significant increase in phenotypic malignant stem cells in the overall HSPC population was observed during the development from MDS to AML. These results revealed a nonlinear, parallel clonal evolution in rare subclones in the progression of MDS to AML. Another study by Stosch et al*.* merged single-cell and bulk sample information to illustrate genetic aberrations, the pertinent clonal architectures, and DNA methylation patterns during the progression of MDS into AML [123]. Single-cell omics has thus matured into a valuable methodology to research disease progression and has provided evidence for subsequent therapy.

JAK2-V617F is the most common mutation in myeloproliferative neoplasm (MPN). Recently, combined with scRNA-seq and mutation detection, Tong et al*.* [124] revealed that JAK2-V617F^+^ HSCs exhibited a bias towards megakaryocyte differentiation. This finding was in accordance with other studies in which megakaryocyte differentiation bias in myelofibrosis [125] and increased frequency of erythroid-megakaryocyte progenitors in MPN [126] were observed. This differentiation bias in MPN indicates that the heterogeneity of stem cells in cancer could help to inform therapeutic guidelines.

Multiple myeloma (MM) is a neoplastic hematologic disorder manifested by a clonal proliferation of malignant plasma cells in the BM [127]. Single-cell transcriptome sequencing can not only study intra- and inter-tumor heterogeneity but also provide new ideas for clinical detection and screening of target drugs [128–130]. Ledergor et al*.* [128] performed scRNA-seq of BM and blood in diagnosed asymptomatic, symptomatic and control individuals to detail the molecular characteristics of MM plasma cells. CD52 was found to enrich circulating tumor cells from peripheral blood, which provided the same or more sensitive genetic information than BM plasma cells. In asymptomatic individuals with early disease, rare tumor plasma cells with molecular characteristics like those of active myeloma could be detected, suggesting that scRNA-seq of early MM may be applied for clinical use. Also, scRNA-seq was reported to detect gene fusions such as t(4;14) in MM [131], implying a new potential application for scRNA-seq.

In conclusion, single-cell omics, combined with integrative bioinformatic analysis, provides new insights into pHSCs, cancer stem cells in hematopoietic malignancies. In spite of this, we usually used mixed samples from different patients in the experiment, it is hard to apply it into individual level. If we want to target some subpopulations or clonal mutations, more meticulous sequencing that personalized to the individual patients is needed. Although it is difficult to apply it into clinical use in a short period of time, that’s making it possible to provide a novel targeted therapy aimed at complex heterogeneous subsets, identify new biomarkers for prediction of prognosis, refine personalized medicine for patients, MRD detection, therapeutic target discovery and even predict response to certain therapies.

### Non-hematological diseases

In human cytomegalovirus (CMV) infection, the molecular mechanisms underlying the latent stage and reactivation still require further research. Through scRNA-seq, it was determined that a small group of CD34^+^ HSPCs expressed markers of Colony Forming Unit—Granulocyte, Erythrocyte, Monocyte, Megakaryocyte (CFU-GEMM) were infected for viral replication [132]. However, another study demonstrated that monocyte progenitors with repressed immune response were the only population in which viral transcripts could be detected in the latent HSPCs. The infection of CMV drives HSPCs towards the weaker immune stage of monocytes, which provides the optimal environment for viral replication [133]. Since 2019, severe coronavirus disease 2019 (COVID-19) became pandemic globally. Interestingly, HSPCs from COVID-19 patients were also impaired due to this highly contagious virus. Through single-cell transcriptome analysis, Wang et al*.* [134] demonstrated that in severe cases, immature myeloid progenitors accumulated and lymphoid progenitors were reduced, and also observed the upregulation of some transcriptome factors (SPI1, LMO4.etc.). Additionally, with the increasing severity of the COVID-19, monocytes showed decreased cell number and weakened response to this disease [135]. Thus, although HSPCs are the basis of hematopoiesis, perturbations in their numbers, differentiation, or temporal dynamics could partly reflect alterations by some non-hematopoietic diseases (e.g., viral infections) or other environmental factors.

### Concluding Remarks

In summary, single-cell omics technologies present a powerful means to reveal the heterogeneity of human HSPCs. Functionally defined, immunophenotyped and transcriptomic-defined HSPCs represent fruitful avenues for future research. The application of single-cell omics to malignant hematopoiesis in recent years has provided new insights into the molecular mechanisms of clonal evolution, disease relapse, and the screening of targeted drugs. Gene and cell therapy areas including hematopoietic stem cell transplantation are important therapies in the treatment of hematologic diseases. However, the intricate operation and high detection cost limit the promotion of single-cell omics. In the future, new simplified single-cell technologies with low cost and high throughput may also lead the way in quality control in the gene and cell therapy arena.

## Abbreviations

HSPCs: Hematopoietic stem and progenitor cells
HSCs: Hematopoietic stem cells
FACS: Fluorescence-activated cell sorting
CB: Cord blood
MPPs: Multipotent progenitors
CMPs: Common myeloid progenitors
MLPs: Multi-lymphoid progenitors
MEPs: Megakaryocyte-erythroid progenitors
GMPs: Granulocyte-monocyte progenitors
scRNA-seq: Single-cell RNA sequencing
MPAL: Mixed-phenotype acute leukemia
AGM: Aorta-gonad-mesonephros
PCW: Post-conception weeks
BM: Bone marrow
HECs: Hemogenic endothelial cells
LMPPs: Lymphoid primed multi-potential progenitors
BMF: Bone marrow failure
AA: Aplastic anemia
DBA: Diamond-Blackfan anemia
PNH: Paroxysmal nocturnal hemoglobinuria
AML: Acute myeloid leukemia
LSCs: Leukemia stem cells
pHSCs: Pre-leukemic HSCs
ALL: Acute lymphoblastic leukemia
T-ALL: T-cell acute lymphoblastic leukemia
BCP-ALL: B cell precursor acute lymphoblastic leukemia
CML: Chronic myeloid leukemia
TKIs: Tyrosine kinase inhibitions
CML-SCs: Chronic myeloid leukemia stem cells
CLL: Chronic lymphocytic leukemia
MDS: Myelodysplastic syndrome
MM: Multiple myeloma
CMV: Cytomegalovirus
